# Accuracy of Wrist-Worn Activity Monitors During Common Daily Physical Activities and Types of Structured Exercise: Evaluation Study

**DOI:** 10.2196/10338

**Published:** 2018-12-10

**Authors:** Ravi Kondama Reddy, Rubin Pooni, Dessi P Zaharieva, Brian Senf, Joseph El Youssef, Eyal Dassau, Francis J Doyle III, Mark A Clements, Michael R Rickels, Susana R Patton, Jessica R Castle, Michael C Riddell, Peter G Jacobs

**Affiliations:** 1 Department of Biomedical Engineering Oregon Health & Science University Portland, OR United States; 2 School of Kinesiology and Health Science York University Toronto, ON Canada; 3 Harold Schnitzer Diabetes Health Center Oregon Health & Science University Portland, OR United States; 4 Harvard John A Paulson School of Engineering and Applied Sciences Harvard University Cambridge, MA United States; 5 Children's Mercy Kansas City Kansas City, MO United States; 6 Institute for Diabetes, Obesity & Metabolism University of Pennsylvania Perelman School of Medicine Philadelphia, PA United States; 7 Department of Pediatrics University of Kansas Medical Center Kansas City, KS United States

**Keywords:** heart rate, energy metabolism, fitness trackers, high-intensity interval training, artificial pancreas

## Abstract

**Background:**

Wrist-worn activity monitors are often used to monitor heart rate (HR) and energy expenditure (EE) in a variety of settings including more recently in medical applications. The use of real-time physiological signals to inform medical systems including drug delivery systems and decision support systems will depend on the accuracy of the signals being measured, including accuracy of HR and EE. Prior studies assessed accuracy of wearables only during steady-state aerobic exercise.

**Objective:**

The objective of this study was to validate the accuracy of both HR and EE for 2 common wrist-worn devices during a variety of dynamic activities that represent various physical activities associated with daily living including structured exercise.

**Methods:**

We assessed the accuracy of both HR and EE for two common wrist-worn devices (Fitbit Charge 2 and Garmin vívosmart HR+) during dynamic activities. Over a 2-day period, 20 healthy adults (age: mean 27.5 [SD 6.0] years; body mass index: mean 22.5 [SD 2.3] kg/m^2^; 11 females) performed a maximal oxygen uptake test, free-weight resistance circuit, interval training session, and activities of daily living. Validity was assessed using an HR chest strap (Polar) and portable indirect calorimetry (Cosmed). Accuracy of the commercial wearables versus research-grade standards was determined using Bland-Altman analysis, correlational analysis, and error bias.

**Results:**

Fitbit and Garmin were reasonably accurate at measuring HR but with an overall negative bias. There was more error observed during high-intensity activities when there was a lack of repetitive wrist motion and when the exercise mode indicator was not used. The Garmin estimated HR with a mean relative error (RE, %) of −3.3% (SD 16.7), whereas Fitbit estimated HR with an RE of −4.7% (SD 19.6) across all activities. The highest error was observed during high-intensity intervals on bike (Fitbit: −11.4% [SD 35.7]; Garmin: −14.3% [SD 20.5]) and lowest error during high-intensity intervals on treadmill (Fitbit: −1.7% [SD 11.5]; Garmin: −0.5% [SD 9.4]). Fitbit and Garmin EE estimates differed significantly, with Garmin having less negative bias (Fitbit: −19.3% [SD 28.9], Garmin: −1.6% [SD 30.6], *P*<.001) across all activities, and with both correlating poorly with indirect calorimetry measures.

**Conclusions:**

Two common wrist-worn devices (Fitbit Charge 2 and Garmin vívosmart HR+) show good HR accuracy, with a small negative bias, and reasonable EE estimates during low to moderate-intensity exercise and during a variety of common daily activities and exercise. Accuracy was compromised markedly when the activity indicator was not used on the watch or when activities involving less wrist motion such as cycle ergometry were done.

## Introduction

### Background

Consumer-based wrist-worn multisensor activity monitors have emerged as an increasingly popular way to track various physiological metrics such as heart rate (HR) and physical activity levels, with the latter being typically expressed in the form of step counts or energy (caloric) expenditure (EE). Sales of activity monitors have doubled from approximately 30 million units in 2014 to approximately 70 million units in 2017 [1,2]. The growth in activity monitors has been largely driven by consumer interest in monitoring and sometimes sharing physical activity levels, workouts, and total daily EE within social networks. In the scientific community, there is increasing interest in whether activity monitors may also be used within a health care setting to collect these same data and help patients and health care providers better manage weight and/or chronic illnesses. For example, in people with type 1 diabetes, aerobic exercise is known to cause steep drops in blood glucose levels, whereas anaerobic exercise can cause glucose levels to rise [3]. Monitoring of patient physical activity levels may be helpful in implementing insulin and/or nutritional strategies to optimize glucose control in type 1 diabetes [4]. In theory, activity monitors can be used in conjunction with on-body continuous glucose monitors, an insulin pump and a control algorithm to adjust insulin delivery, and perhaps glucagon delivery in real time [5,6]. Activity monitors can also be used within algorithm-driven decision support systems to help avert exercise-induced hypoglycemia or late onset hypoglycemia. Automated insulin delivery systems can potentially modify insulin dosing in response to activity monitors to reduce the risk (or severity) of exercise-induced hypoglycemia in people living with type 1 diabetes [7-10]. For any medical system utilizing an activity monitor, the accuracy of the HR and EE estimates by the activity monitor is critical as it can influence medical dosing decisions and patient outcomes. There are 3 distinct challenges with using the activity monitors within medical systems, namely, detecting the onset of the activity, distinguishing the type of the detected activity, and estimating the intensity and duration of the activity, as each of these functions can determine how medical systems may behave. In this paper, we explore the accuracy of HR and EE estimates from 2 popular activity monitors to determine if the accuracy of these wearables is sufficient for use within medical applications such as automated insulin delivery systems for use within type 1 diabetes glucose management.

In the earlier models of activity monitors, only accelerometers were used to estimate EE [11], but in more recent multisensor models, photoplethysmography (PPG) is being used to estimate HR [12] and, potentially, to improve the accuracy in estimating EE [13]. With the inclusion of HR as measured by the PPG sensor and acceleration as measured by the accelerometer, the accuracy of the estimated EE is expected to be improved in newer models. For example, Zakeri et al [14] showed that EE can be estimated using both accelerometry and HR along with several additional patient-specific parameters such as age, weight, and height. The Zakeri et al algorithm utilizing accelerometry and HR to estimate EE and metabolic equivalents (METs) has been used in the past to inform an automated insulin delivery system during physical exercise [6]. In a post hoc analysis that combined both HR and accelerometer signals, researchers demonstrated that steady-state aerobic exercise could be detected early before rapid changes to glucose occurred [15]. In recent studies involving predominantly steady-state aerobic activities, wrist-worn activity monitors have been shown to have reasonable accuracy in HR estimation (approximately 5% error) but a poor estimate of EE, where the error was found to be closer to approximately 30% with a negative bias [16,17]. In free-living conditions, however, activity monitors are worn typically on the nondominant wrist during multiple forms of exercise in nonsteady states, not just aerobic exercise on a treadmill performed at a constant workload or intensity (steady-state). For example, in free-living conditions, many individuals often perform resistance exercise using free weights or their own body weight, followed by some form of high-intensity interval training (HIIT) within the same session. In fact, in the diabetes population, patients are encouraged to perform both resistance and aerobic training all in one session. HIIT has recently been recommended to rapidly improve fitness, body composition, and overall glycemic control [18-20].

Presently, there are at least 4 studies [21-24] that have investigated the accuracy of wearable devices during resistance exercises and none during HIIT training. Bai et al [21] reported that EE measured during an unstructured resistance exercise protocol in which participants selected exercises and loads was inaccurate across numerous devices. The devices included 5 wrist-worn devices (Fitbit Flex, Jawbone Up24, Misfit Shine, Nike+ Fuelband SE, and Polar Loop) and 2 research monitors (Actigraph GT3X+ on the waist and the BodyMedia Core on the arm). In this study, 52 participants tested these 7 different devices, and the wearable devices had lower accuracy for EE when compared with a metabolic analysis system. None of the devices in this study reported HR measures. Horton et al [22] assessed the validity of HR only using the Polar M600 when compared with a 3-lead electrocardiogram (ECG) during both aerobic and resistance exercises. The accuracy of the wearable device was reported to be better during aerobic exercise (92%) as compared with only 35% accurate during the resistance exercises. In this study, participants completed squats, shoulder shrugs, bicep curls, and lunges with dumbbells at a self-selected weight. Jo E et al [23] reported poor correlation and HR accuracy in the Fitbit Charge HR device. In this study, subjects completed a short-resistance exercise bout involving resisted arm raises, resisted lunges, and isometric plank. In a large cohort study, Bourdreaux et al [24] standardized the selection of the weights utilized during the resistance exercises: 2 upper body exercises (chest press, latissimus dorsi pulldown) and 2 lower body exercises (leg extension and leg curl) among the subjects using a standardized 10-rep max protocol. Results from this study demonstrated that HR measured by nonwrist worn devices were relatively accurate, whereas wrist-worn devices showed poor correlations (*R*<.8) and higher error during resistance exercises (mean absolute percent error [MAPE] >9%). They also showed that the EE measured by the devices was poor, with MAPE values ranging between 43% and 57%.

### Objectives

The primary aim of this study was to examine the accuracy of both HR and EE across a wide range of dynamic activities including resistance training, HIIT, and aerobic training. A secondary aim was to examine the accuracy when the optional *activity mode* is not selected on the wearable. There may be times when people exercise, but they do not indicate that they are exercising; we wanted to determine the accuracy both when they do and do not indicate that they are exercising.

## Methods

### Participants

The experimental protocol conformed to the standards set by the Declaration of Helsinki and was approved by the institutional review board at the Oregon Health and Science University (OHSU, Portland Oregon) and by the research ethics board at York University (Toronto, Canada). This study recruited 20 healthy adults (11 females; 10 subjects at OHSU; 10 at York University) who all provided informed consent before taking part in the study. Participants were screened for any cardiovascular complications using a Physical Activity Readiness Questionnaire [25].

### Study Protocol

Participants attended the research laboratory on 2 separate occasions, separated by 24 hours. Each visit involved simultaneous recordings of HR (beats per minute) and EE (kcals and METs) from the respective criterion measures during a series of physical activities and structured exercises. On the first visit, a stadiometer (Seca, model220, Hamburg, Germany) was used to measure height to the nearest 0.25 cm (without shoes) and body mass was measured to the nearest 0.1 kg using a scale (Seca, model 707, Hamburg, Germany), with the participant dressed in workout clothes. As per the manufacturer’s instructions, age, gender, height, and weight were used to initialize the wearable devices and associated applications. These same data were also inputted to a portable metabolic unit (Cosmed, Rome, Italy). Two wearable devices (one of each brand) were tested at the same time on all participants (one on each wrist as per manufacturer’s instructions) using a randomized and counterbalanced method. On each visit, participants undertook 2 activity blocks (see below for further details) following setup of the devices and synchronization of all the devices to a single clock before the exercise protocol commenced.

### Activities

At visit 1, participants performed 2 blocks of physical activity separated by a 30-min rest period. In the first block, participants performed a graded maximal aerobic exercise test (treadmill or cycle ergometer, 10 subjects per mode) to volitional exhaustion (ie, progressive to peak oxygen consumption, VO_2_ peak). These will be referred to as MAX-T (MAX-treadmill) and MAX-C (MAX-cycle ergometer) tests. During MAX-T, each participant began with a 5-min standing rest, followed by 4 min of walking as a warm-up (3.0 mph, 0% grade for 2 min then at 5% grade for 2 min). After the warm-up, participants self-selected a comfortable running speed between 4 to 6 mph, and subsequently, the treadmill incline was increased by 2% every 2 min until the participant reached volitional exhaustion. At each workload stage, participants were asked to assess their level of physical exertion using the Borg Rating of Perceived Exertion (RPE) 10-point scale [26]. For the participants performing the MAX-C test, each participant began with a 5-min seated rest followed by 4 min of warm-up cycling at a moderate cadence (approximately 50-60 revolutions per minute [rpm]) at zero load. After this, cycling cadence was maintained at 60 rpm, and the power output was increased every 2 min by 30 watts until the participant reached volitional exhaustion. Borg RPE was assessed at the end of each 2-min stage. For both MAX-T and MAX-C protocols, the wearables were placed in the appropriate exercise setting (ie, running or cycling) and worn on the wrist as per manufacturer’s specifications. Following the exercise test, the participants rested for 30 min. In the second block of activity on the same day, a resistance circuit workout was performed (2 sets of 8 repetition max of all the major muscle groups). Subjects selected a suitable dumbbell weight that they could maintain a proper form for 8 repetitions before muscular fatigue. The following 6 exercises were performed: dumbbell bicep curls, Romanian deadlifts, Bulgarian split squat, dumbbell bench press, dumbbell shoulder press, and dumbbell step ups. After a 20-min cool-down, participants then left the laboratory.

At visit 2, performed the next day, participants undertook 2 new activity blocks. The first activity block consisted of 28 min of routine activities of daily living (ADLs), while the second block included high-intensity interval training (HIIT) for 27 min (including warm-up and cool-down). Six ADLs were performed to simulate daily chores. Each activity was 3 min in duration. Activities included sitting on a chair or lying on a bed, washing of dishes and simulated loading and unloading of a dishwasher, sweeping or vacuuming of a small room, organizing a room or adjusting furniture in the room, scrubbing of walls and carpet/floor, and self-paced ascending and descending of a flight of stairs. These activities were preceded and followed by two 5-min segments of seated rest. In the second activity block, participants executed the same exercise mode (ie, treadmill and cycle ergometer) as was done in the peak exercise test. The high-intensity activities are referred to as HIIT-T (HIIT-treadmill) and HIIT-C (HIIT-cycle ergometer). For HIIT-C, participants were asked to cycle at approximately 60 rpm for 2 min at a low intensity with low resistance, corresponding to approximately 30% of their peak power output in watts (as measured during MAX-C), and then at a high intensity (60 rpm), at a power output corresponding to approximately 80% of their peak power output for 2 min, for a total of 5 cycles. For the treadmill intervals, participants were asked to walk for 2 min at a treadmill speed and slope corresponding to approximately 30% HR reserve (as measured during MAX-T), and then run/jog at a speed and slope corresponding to approximately 80% of their HR reserve for 2 min, for a total of 5 cycles. This session was completed following a cool-down period of 5 min.

### Wearables Devices

Although multiple devices were available that could provide the relevant exercise metrics, we chose the following 2 devices mentioned below after considering their costs and their ability to integrate with a control system running on an Android platform. Henriksen et al provided a detailed review of the many devices that are available and have been tested over the last few years [27].

### Garmin vívosmart HR+

The Garmin vívosmart HR+ (2016 version, Garmin International Inc, Kansas, US) is a multisensor activity monitor that has an accelerometer, global positioning system, and built-in PPG sensor that uses the “Elevate” wrist HR technology to measure HR at the wrist. According to the device specifications, the frequency at which HR is measured is normally once every 15 seconds, but triggering the *device key* button and setting the wearable to an activity mode (eg, run) increases the frequency at which HR is measured. EE values are reported in calories for a given activity session, also when the device key is pressed. Garmin provided a special interface to export data from the device when the *device key* button was not indicated. This provided a reliable method to download data. The firmware version of the device was 3.20. Data were exported via Bluetooth low energy (BTLE) to the Garmin-Connect App version 3.17.

### Fitbit Charge 2

The Fitbit Charge 2 (2017 version, Fitbit Inc, California, US) is a multisensor activity monitor that has an accelerometer and built-in PPG sensor that uses the “PurePulse” wrist HR technology to measure HR at the wrist. The sample rate at which HR is measured varies and depends on the level of activity; the Charge 2 uses SmartTrackTM to automatically detect and record select exercises, but the manufacturer recommends using the exercise menu to improve the precision of HR and EE measurements. All data collected from the Fitbit were collected with the particular exercise selected as recommended by the manufacturer. Data could not be exported reliably without the type of exercise selected via the button press; this prevented the collection of data without the users pressing the button indicating the type of activity selected. According to the manufacturer, the frequency at which HR is measured during activity mode is once every second. EE values are reported in calories for a given exercise session. Data were exported via BTLE to the Fitbit App version 2.35. The firmware version of the device was 22.54.6. Data were downloaded at the highest sample rate possible through Fitabase (Small Steps Labs, California, US), a third party research platform designed to collect data from Fitbit using the developer application programming interface (API). The use of Fitbit with Fitabase also allows for estimates of METs for an additional assessment of the relative energy costs of a given activity, compared with rest, and for the determination of estimated oxygen consumption (VO_2_) expressed in ml O_2_·kg^−1^·min^−1^.

### Heart Rate Criterion Measure

Participants wore the Polar H7 (BTLE version, Polar Electro, Kempele, Finland) chest strap HR monitor, which was secured tightly to ensure skin contact. The data from the Polar H7 was transmitted to the Polar A300 (Polar Electro, Kempele, Finland), and the second level data from this device was downloaded using the Polar Flow App. Although some studies have shown the limitation of these devices as compared with the gold standard ECG measure of HR [24,28], these chest-based HR monitors have been used to inform glucose control systems of exercise [8,10,15].

### Energy Expenditure Criterion Measure

Participants wore a portable indirect calorimeter, Cosmed K4b2 or Cosmed K5 (Rome, Italy), which collected breath-by-breath data on the ventilatory parameters (ie, oxygen consumption, VO_2_). EE was estimated from the direct measurement of oxygen consumption and carbon dioxide production. The units were calibrated before each session according to the manufacturer’s instructions. EE data were downloaded from the cardiopulmonary exercise testing suite.

### Statistical Analysis

Statistical analysis was performed separately for HR and EE. Data from the indirect calorimetry (VO_2_ and carbon dioxide consumption [VCO_2_]) served as the reference standard measurement for calculations of EE (kcal/min). Data from the Polar HR monitor served as the as the reference standard for HR (beats per minute, bpm). In this analysis for both EE and HR, we analyzed all the data collected from each device, and error was calculated as device measurement-reference standard, and mean relative error (RE, %) was calculated as the mean of the device measurement-reference standard × 100/reference standard. We also report MAPE as the mean of the absolute value of device measurement-reference standard × 100/reference standard. Error in HR was calculated at each measurement using the closest data collected from the reference standard as the reference measurement. We observed in our data that the sample rate of the devices varied, with the reference standard Polar measuring the HR every second, the Fitbit measuring every 1 to 15 seconds, and Garmin measuring every 5 to 60 seconds. Pearson (*r*) correlation coefficient and Bland-Altman analysis were used to assess the mean bias and agreement between the devices and the reference standard. We adopted the widely accepted level of accuracy of 5% to be within the acceptable limits [16]. Student *t* test with the Satterthwaite approximation was performed to assess the difference in HR measured between Garmin devices when the activity mode was indicated and when it was not. We also performed the same statistical tests to assess the differences between the errors in the HR measurements for activities with repetitive wrist motion (treadmill tests) as compared with activities with no repetitive wrist motion (ergometer tests). Error in EE was only calculated across an entire activity session as higher resolution data could not be obtained from the devices. Matched paired *t* tests were performed to assess the difference in RE and MAPE of EE between Fitbit and Garmin for each activity. One-way analysis of variance with a Tukey honest significant difference post hoc test was performed to assess the difference in RE and MAPE of EE between activities within each device. We used concordance class correlation to measure agreement between the devices tested. All statistical analyses were conducted in R (R Core Team, Vienna, Austria, version 3.4.2) and GraphPad Prism 7 (GraphPad Software, La Jolla, CA, version 7.0c) [29].

## Results

### Cohort

All 20 participants recruited for the study completed the procedures. Table 1 describes the participant characteristics.

### Heart Rate Accuracy

**Figure 1:**
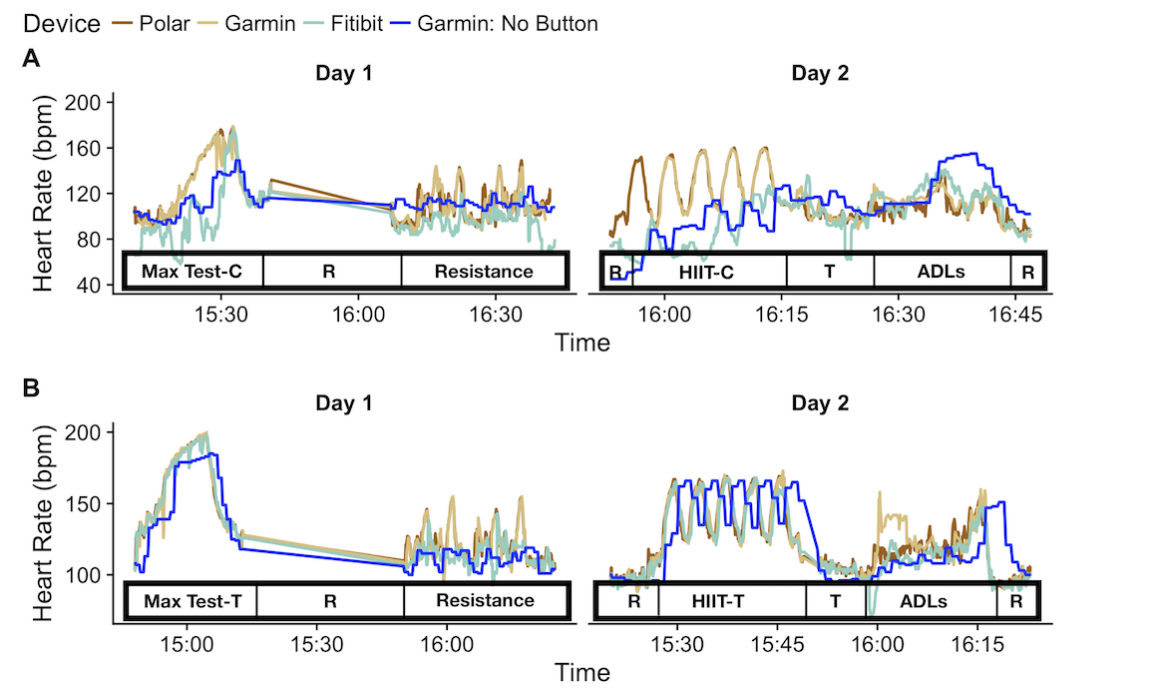
Two-day study protocol with “R” indicating the rest periods and “T” indicating the transition period between the different types of activities. Data are shown from 2 different participants wearing all devices in panels A and B. Note, Garmin devices were worn by the participants here in 2 different modes: one with the activity mode indicated (Garmin) and the other without (Garmin: No Button). Panel A shows the data during the cycle ergometer tests and panel B shows the data from the treadmill tests. Data in panel A highlight the error observed during higher intensity exercises where wrist movement was less pronounced during cycle ergometer testing. Panel B shows treadmill results when the Garmin, Fitbit, and Polar data are very closely matched across the exercise types. ADLs: activities of daily living; C: cycle ergometer; HIIT-C: high-intensity interval training-Cycle ergometer; HIIT-T: high-intensity interval training-Treadmill; T: treadmill.

We analyzed a total of 83,349 simultaneous HR pairs of data, whereby a pair is either a Garmin or a Fitbit measurement compared with the reference standard (Polar chest strap). There were a total of 61,499 pairs for the Fitbit HR data, 18,317 pairs of HR data from Garmin (with the activity mode indicated), and 3533 pairs of HR data from Garmin with no button press (activity mode not indicated). We analyzed data collapsed across all activities and also looked at accuracy during each individual activity. There was no difference in accuracy between the 2 devices when the activity mode was indicated. The overall performance was significantly worse if the activity mode was not indicated on the Garmin device compared with when activity mode was indicated (*P*<.001). Figure 1 shows results of the HR data across a test session for 1 subject. Both panels show that when the activity mode is not initiated on the wearable, there is less accuracy and also a distinct phase shift whereby the Garmin with no button trace appears to be shifted in time relative to the Polar. This shift in time is a minor contributor to the inaccuracy within the HIIT activities. The majority of error was from devices failing to track during dynamic activities.

For HR data collected with the activity mode indicated, a systematic negative bias was observed in both Fitbit and Garmin devices. The mean relative error, RE (SD) for the Fitbit device on the collapsed data was −4.71% (19.63), the mean RE (SD) for the Garmin (with activity mode indicated) was −3.33% (16.67), and the mean RE (SD) for the Garmin (with activity mode not indicated) was −5.47% (22.79; comparing the Garmin devices with activity mode indicated vs not indicated. *P*<.001). MAPE (SD) for the Garmin and Fitbit was 10.79 % (13.14) and 11.33% (16.71), respectively. Mean HR accuracy across each activity was analyzed and compared with the reference standard; these data are shown in Table 2.

**Figure 2:**
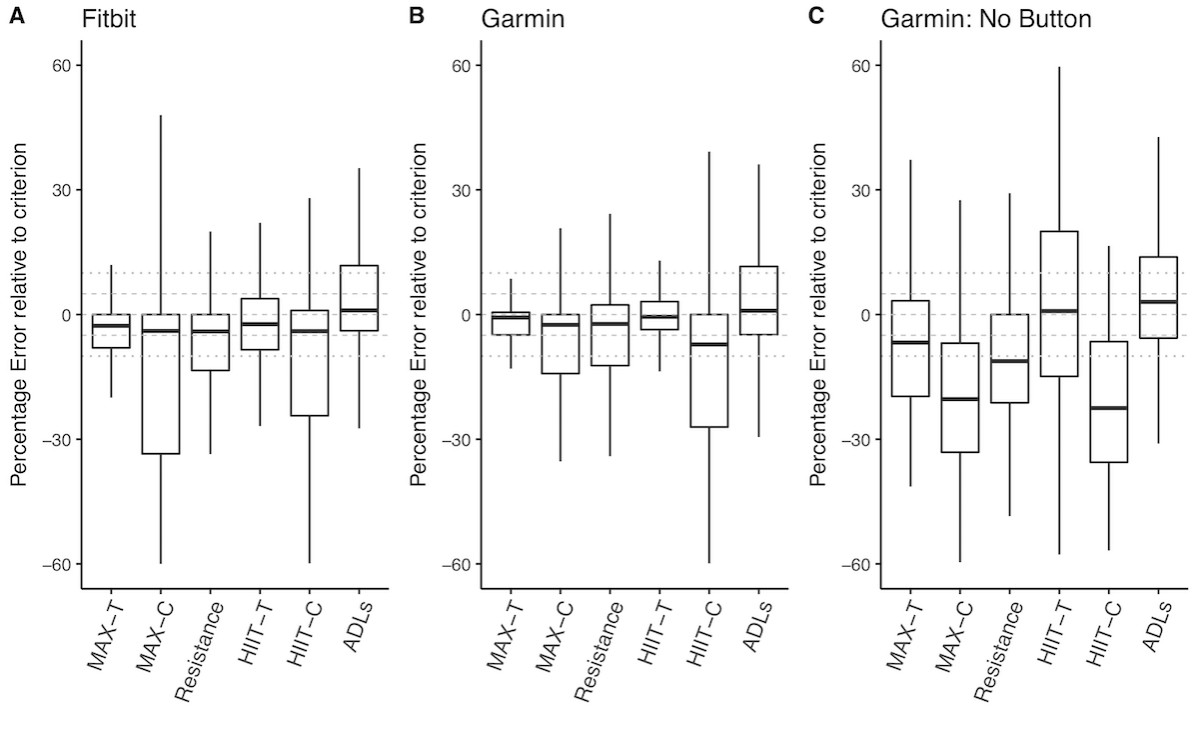
Percent relative error (RE) in heart rate (HR) across all the activities from all the devices tested. Percent error is calculated as (device measurement-reference standard) × 100/reference standard. The box-whisker plots indicate the error with the 25% quartile, median (50% quartile), and 75% quartile marked in each box plot. Gray horizontal dashed lines indicate the 5% error threshold, and the dotted lines indicate the 10% error threshold. ADLs: activities of daily living, HIIT-C: high-intensity interval training-Cycle ergometer, HIIT-T: high-intensity interval training-Treadmill, MAX-C: MAX-Cycle ergometer, MAX-T: MAX-Treadmill.

The lowest mean error in measuring HR was observed during the HIIT-T (Fitbit: −1.7% [SD 11.5], Garmin: −0.5% [SD 9.4]), whereas the highest error was observed on both HIIT-C (Fitbit: −11.4% [SD 35.7], Garmin: −14.3% [SD 20.5]) and during MAX-C (Fitbit: −16.4% [SD 21.6], Garmin: −9.3% [SD 17.0]). Figure 2 shows the variability between and within activities. When the activity mode of the wearables are activated (panels A and B), median % relative errors are within the 5% error threshold for both devices. When the activity mode is not activated, as observed in panel C, the median % relative error significantly exceeds the 5% threshold across many of the activities.

**Figure 3:**
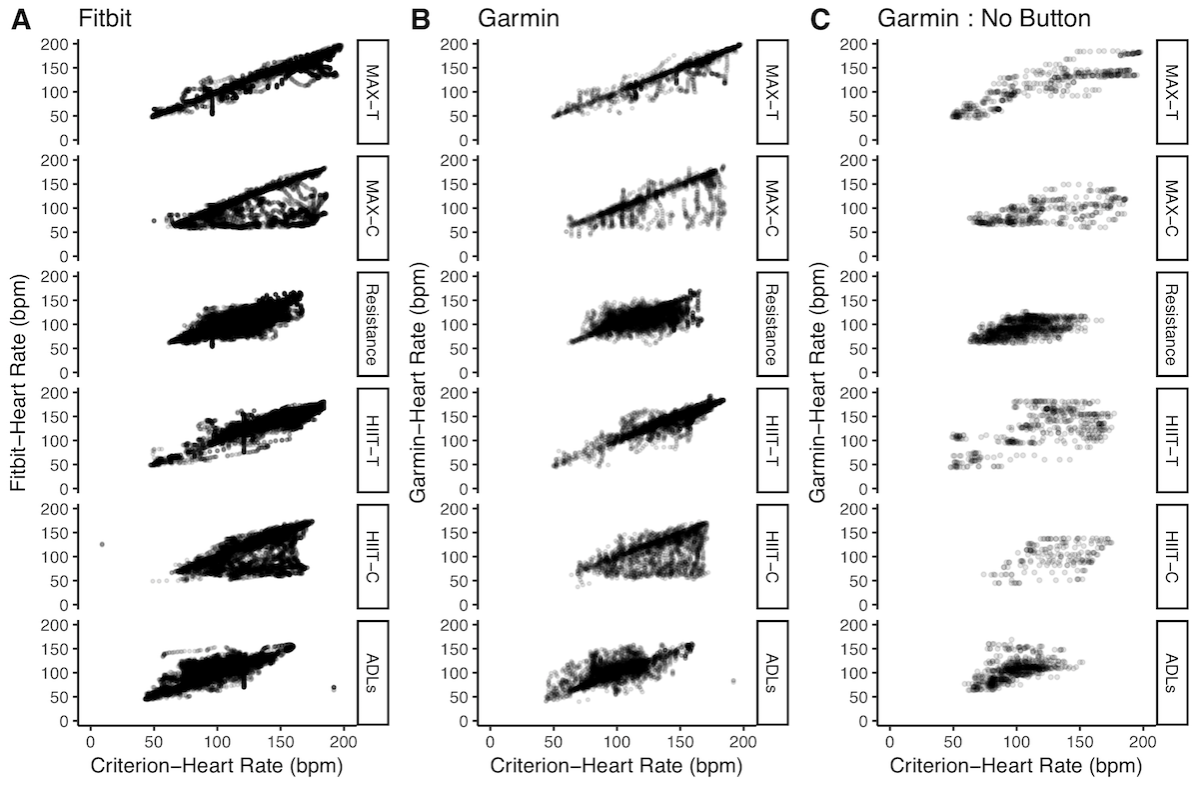
Scatter plots showing HR measurements from Fitbit and Garmin versus the reference standard Polar across all activities. Panel A shows the correlation plot comparing the Fitbit versus the Polar. Panel B shows the correlation plot comparing the Garmin (with activity mode indicated) versus the Polar. Panel C shows the correlation plot for a subset of the subjects comparing the Garmin (with activity mode not indicated) versus the Polar. ADLs: activities of daily living, HIIT-C: high-intensity interval training-Cycle ergometer, HIIT-T: high-intensity interval training-Treadmill, MAX-C: MAX-Cycle ergometer, MAX-T: MAX-Treadmill.

The correlation between the HR values on the wearables and our gold standard chest band sensor was best during MAX-T (Fitbit: 0.94, Garmin: 0.94), whereas poor correlation between the HR values was observed during the HIIT-C (Fitbit: 0.46, Garmin: 0.71). The relative error across the collapsed data for the activities with repetitive motion of the upper torso (ie, treadmill tests) was observed to be significantly lower at −1.6% (SD 9.6) when compared with activities with no repetitive motion of the upper torso (ie, cycle ergometer tests) at −12.25% (SD 19.3; *P*<.001). Scatter plots between the simultaneous measures across all the activities are shown in Figure 3.

**Figure 4:**
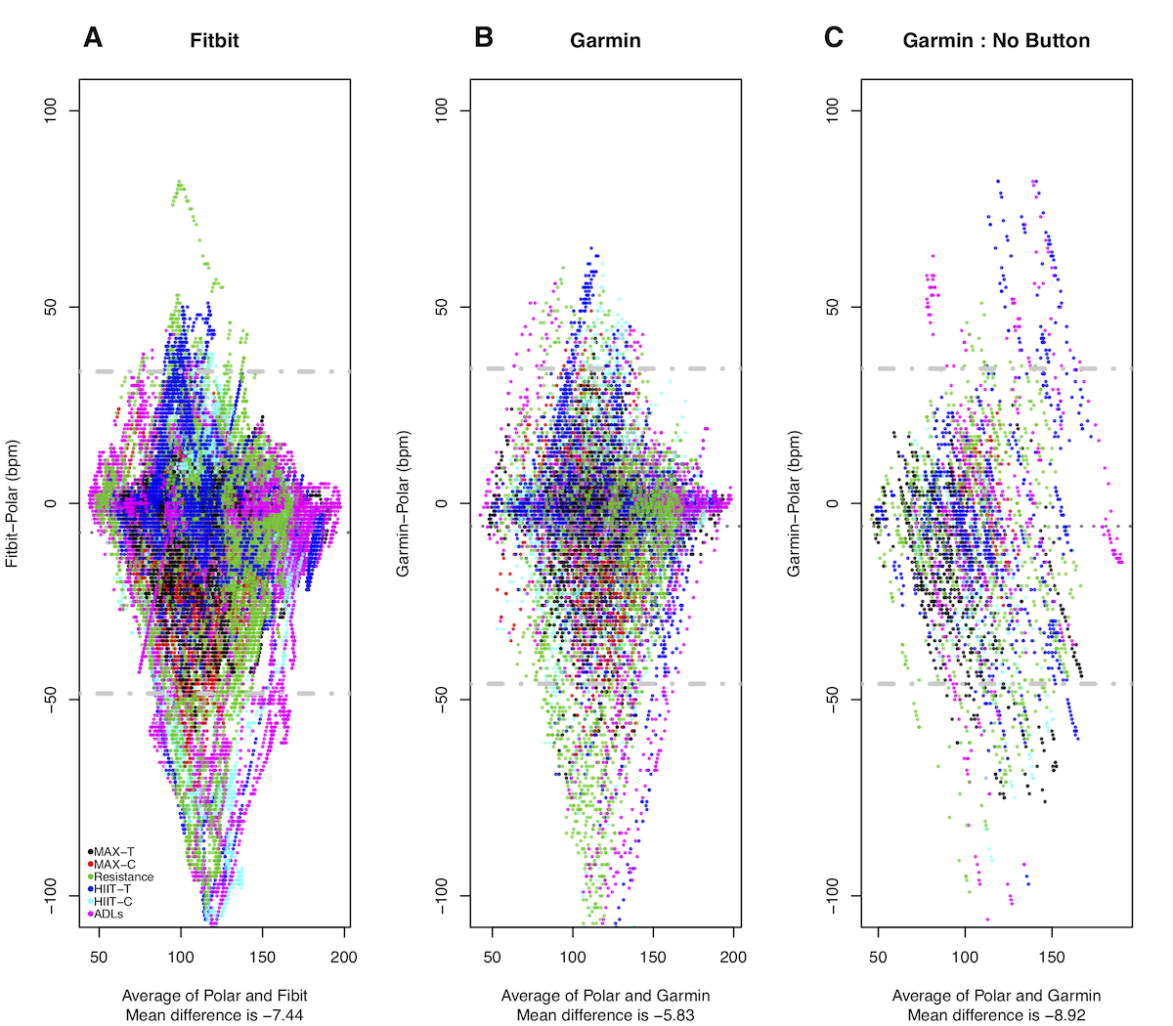
Bland-Altman plots showing heart rate measurements comparing Garmin and Fitbit relative to Polar for all data with activities indicated by color. Mean heart rate is shown on the x-axis, and the difference between the Garmin or Fitbit and the Polar heart rate is on the y-axis. The gray dotted line indicates the mean difference (bias) between the measurement, and the gray dashed lines indicate the limits of agreement. Panel A compares the Fitbit and the Polar. Panel B compares the Garmin (with activity indication) and the Polar. Panel C compares the Garmin (with no activity indication) and the Polar.

Bland-Altman plots indicated that all 3 devices underestimated the HR when compared with the reference standard as indicated in Figure 4. The variability between these devices was comparable. However, the wearable devices tended to have significantly higher error when the HR signal transitioned quickly and at higher intensity.

There was a generally small but significant impact of the wrist side worn (ie, left vs right) on the percent absolute relative error. Using a *t* test, the error was shown to be higher on the right hand versus the left hand for the MAX-T (6.6% vs 5.1%, *P*<.001), HIIT-T (6.72% vs 5.85%, *P*=.002), and ADLs (13.33% vs 11.17%, *P*<.001), whereas the error was higher on the left hand versus the right hand for resistance (15.0% vs 13.5%, *P*<.001) and MAX-C (9.53 vs 2.97%, *P*<.001).

### Energy Expenditure Accuracy

Due to the limitation on the Garmin Connect application, EE data could only be compared at a low resolution, namely an average across each activity mode (eg, ADL, HIIT-C, or HIIT-T). Both Fitbit and Garmin performed reasonably well in estimating task-specific EE, when looking at the group as a whole, but considerable error was noted for some of the activities, particularly with cycling activities for Fitbit and resistance activities for Garmin. Fitbit and Garmin EE estimates differed significantly, with Garmin having less negative bias overall (Fitbit: −19.3% [SD 28.9], Garmin: −1.6% [SD 30.6]; *P*<.001). Table 3 shows the error in EE estimations for each of the activities for both devices.

**Figure 5:**
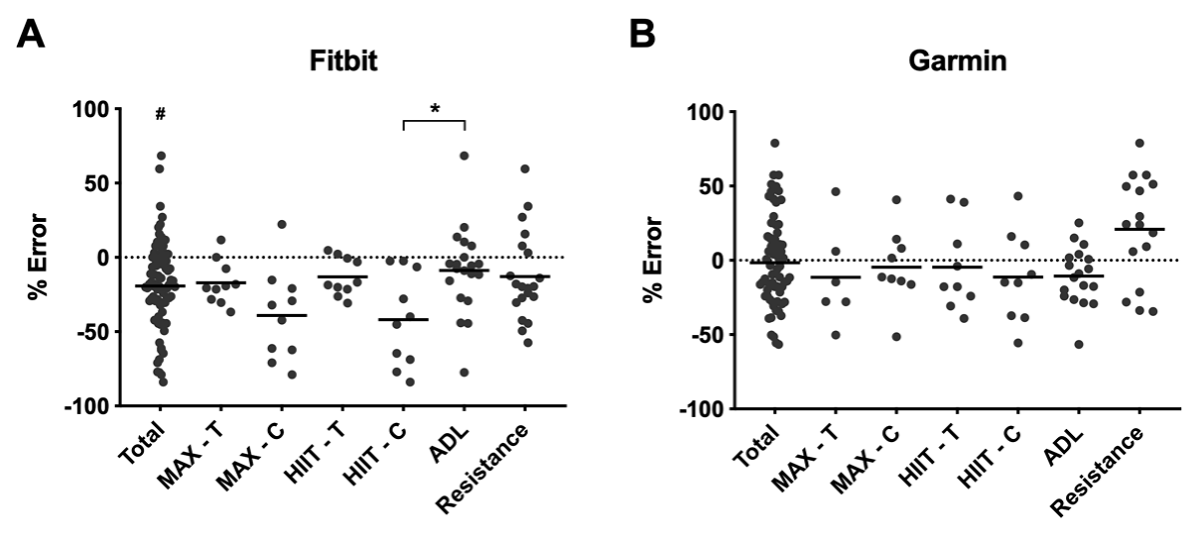
Percent relative error (RE) in energy expenditure (EE) across different exercise modalities for Fitbit (A) and Garmin (B). Negative bias in estimating EE is apparent across exercise modalities. The horizontal lines represent the mean. Asterisk indicates *P*=.03; # indicates *P*<.001 compared to Garmin. ADLs: activities of daily living, HIIT-C: high-intensity interval training-Cycle ergometer, HIIT-T: high-intensity interval training-Treadmill, MAX-C: MAX-Cycle ergometer, MAX-T: MAX-Treadmill.

Figure 5 shows the % relative error (RE) in EE for Fitbit and Garmin during each activity as scatter plots, when compared with the Cosmed indirect calorimeter.

**Table 1:** Participant characteristics (n=20). VO_2_ max (maximal oxygen uptake) was measured at the incremental test to exhaustion.

Characteristic		Value
Age (years), mean (SD)		27.5 (6.0)
Height (cm), mean (SD)		173.2 (9.5)
Weight (kg), mean (SD)		67.9 (10.8)
Body mass index (kg/m^2^), mean (SD)		22.5 (2.3)
VO_2_ max (mL/min/kg), mean (SD)		48.0 (8.7)
Wrist (cm), mean (SD)		15.6 (2.0)
**Race, n (%)**		
	White	17 (85)
	Asian	2 (10)
	Native American/Canadian	1 (5)

**Table 2:** Heart rate accuracy data across all subjects for the different activity types undertaken during the study: sample size, mean (SD) of each of the measured devices, mean (SD) of the difference between the device measurement and the reference standard, the mean relative difference (SD; %), the mean absolute difference (SD; %), and the correlation between the measures.

Heart rate (beats per minute) and measures				Fitbit		Garmin		Garmin + no button
**Max test (treadmill): progressive exercise to volitional fatigue**								
		Pairs, N	7127		2037		476	
		Device, mean (SD)	129.6 (38.0)		139.6 (37.3)		112.2 (38.2)	
		Criterion, mean (SD)	137.2 (40.9)		144.7 (36.5)		122.3 (45.5)	
		Mean difference (SD)	−7.6 (13.6)		−5.1 (13.0)		−10.1 (21.5)	
		% mean relative error (SD)	−4.8 (10.3)		−3.3 (9.6)		−5.9 (16.6)	
		% mean absolute error (SD)	7.3 (11.8)		5.8 (8.4)		14.5 (10.1)	
		Concordance class correlation (95% CI)	0.92 (0.92-0.93)		0.93 (0.92-0.93)		0.84 (0.82-0.87)	
		Pearson correlation	.94		.94		.88	
**Max test (ergometer): progressive exercise to volitional fatigue**								
	Pairs, n			6375		1705		444
	Device, mean (SD)			101.4 (31.2)		115.5 (34.0)		91.5 (21.3)
	Criterion, mean (SD)			125.3 (32.7)		128.9 (33.3)		120.3 (34.1)
	Mean difference (SD)			−23.8 (33.4)		−13.4 (25.6)		−28.8 (27.8)
	% mean relative error (SD)			−16.4 (21.6)		−9.3 (17.0)		−20.6 (18.2)
	% mean absolute error (SD)			17.9 (32.3)		11.8 (15.3)		22.9 (15.2)
	Concordance class correlation (95% CI)			0.36 (0.34-0.37)		0.66 (0.62-0.68)		0.34 (0.29-0.39)
	Pearson correlation			.46		.71		.58
**Resistance exercise**								
		Pairs, n	17,420		5215		1200	
		Device, mean (SD)	105.9 (21.2)		112.9 (17.7)		91.8 (15.6)	
		Criterion, mean (SD)	114.4 (21.4)		119.5 (20.1)		104.6 (19.4)	
		Mean difference (SD)	−8.5 (14.4)		−6.5 (17.5)		−12.8 (17.4)	
		% mean relative error (SD)	−6.9 (12.0)		−4.2 (14.2)		−10.7 (14.9)	
		% mean absolute error (SD)	9.8 (12.1)		10.6 (10.4)		15.0 (10.7)	
		Concordance class correlation (95% CI)	0.72 (0.71-0.72)		0.54 (0.52-0.56)		0.4 (0.37-0.45)	
		Pearson correlation	.88		.9		.53	
**Daily chores and activities of daily living**								
		Pairs, n	14,883		3605		738	
		Device, mean (SD)	101.8 (20.5)		104.0 (22.0)		104.5 (20.8)	
		Criterion, mean (SD)	98.6 (20.8)		100.2 (21.8)		98.2 (17.0)	
		Mean difference (SD)	3.3 (15.2)		3.9 (17.4)		6.3 (18)	
		% mean relative error (SD)	3.3 (16.50)		5.6 (19.5)		7.4 (19.4)	
		% mean absolute error (SD)	11.4 (11.2)		13.0 (13.2)		14.0 (15.4)	
		Concordance class correlation (95% CI)	0.72 (0.71-0.73)		0.68 (0.66-0.69)		0.52 (0.47-0.57)	
		Pearson correlation	.73		.69		.56	
**Treadmill: intermittent high-intensity exercise**								
		Pairs, n	8105		3315		482	
		Device, mean (SD)	129.7 (28.0)		138.8 (26.9)		125.7 (38.1)	
		Criterion, mean (SD)	133.2 (30.6)		139.9 (26.3)		120 (35.4)	
		Mean difference (SD)	−3.5 (14.4)		−1.2 (11.9)		5.7 (33.5)	
		% mean relative error (SD)	−1.7 (11.5)		−0.5 (9.4)		8.9 (33)	
		% mean absolute error (SD)	8.5 (10.0)		9.0 (6.0)		25.0 (23.3)	
		Concordance class correlation (95% CI)	0.87 (0.87-0.88)		0.90 (0.89-0.91)		0.58 (0.52-0.63)	
		Pearson correlation	.88		.9		.59	
**Ergometer: intermittent high-intensity exercise**								
		Pairs, n	7589		2440		193	
		Device, mean (SD)	110.6 (31.2)		110.9 (30.3)		100.4 (26.6)	
		Criterion, mean (SD)	127.0 (25.7)		131.2 (25.3)		131.2 (24.2)	
		Mean difference (SD)	−16.4 (27.2)		−20.3 (28.9)		−30.8 (27.4)	
		% mean relative error (SD)	−11.4 (35.7)		−14.3 (20.5)		−22.5 (19.8)	
		% mean absolute error (SD)	16.0 (24.4)		26.0 (17.6)		25.0 (13.4)	
		Concordance class correlation (95% CI)	0.47 (0.45-0.48)		0.37 (0.34-0.39)		0.24 (0.16-0.32)	
		Pearson correlation	.56		.47		.42	

**Table 3:** Pooled energy expenditure data for the different types of activities undertaken during the study. Data are shown for each activity type. Sample size, mean (SD) of each of the measured device, mean (SD) of the difference between the device measurement and the reference standard, the mean relative difference (SD; %), the mean absolute difference (SD; %), and the correlation between the measures.

Energy expenditure (kcal) and measures		Fitbit		Garmin	
**Max test (treadmill): progressive exercise to volitional fatigue**		**N=10**		**N=6**	
	Device, mean (SD)		192.1 (47.2)		216.5 (55.3)
	Criterion, mean (SD)		237.3 (72.5)		260.5 (77.2)
	Mean difference (SD)		−45.2 (44.4)		−44.0 (90.1)
	% mean relative error (SD)		−17.0 (14.6)		−11.4 (33.7)
	% mean absolute error (SD)		19.4 (11.0)		28.8 (17.2)
	Pearson correlation		.81		.11
**Max test (ergometer): progressive exercise to volitional fatigue**		**N=10**		**N=9**	
	Device, mean (SD)		133.6 (77.6)		207.0 (48.7)
	Criterion, mean (SD)		225.3 (74.7)		231.4 (76.5)
	Mean difference (SD)		−91.7 (87.2)		−24.4 (63.9)
	% mean relative error (SD)		−39.1 (30.6)		−4.5 (25.3)
	% mean absolute error (SD)		43.5 (23.0)		18.9 (16.2)
	Pearson correlation		.35		.56
**Resistance exercise**		**N=20**		**N=16**	
	Device, mean (SD)		130.2 (46.2)		179.8 (56.8)
	Criterion, mean (SD)		153.1 (45.5)		155.2 (47.8)
	Mean difference (SD)		−22.9 (44.0)		24.6 (56.6)
	% mean relative error (SD)		−12.9 (29.7)		21.0 (35.7)
	% mean absolute error (SD)		27.7 (15.9)		35.7 (19.7)
	Pearson correlation		.54		.43
**Daily chores and activities of daily living**		**N=20**		**N=18**	
	Device, mean (SD)		103.5 (38.2)		100.6 (23.4)
	Criterion, mean (SD)		114.4 (25.7)		114.8 (27.0)
	Mean difference (SD)		−10.9 (39.4)		−14.3 (28.2)
	% mean relative error (SD)		−8.8 (29.2)		−10.6 (19.3)
	% mean absolute error (SD)		20.9 (21.8)		17.0 (13.7)
	Pearson correlation		.29		.38
**Treadmill: intermittent high-intensity exercise**		**N=10**		**N=9**	
	Device, mean (SD)		211.1 (57.0)		226.9 (58.1)
	Criterion, mean (SD)		246.6 (71.9)		249.7 (75.6)
	Mean difference (SD)		−35.5 (34.6)		−22.8 (61.7)
	% mean relative error (SD)		−13.1 (12.7)		−4.7 (29.3)
	% mean absolute error (SD)		14.5 (10.9)		25.0 (3.4)
	Pearson correlation		.88		.60
**Ergometer: intermittent high-intensity exercise**		**N=10**		**N=9**	
	Device, mean (SD)		128.2 (60.4)		205.8 (76.4)
	Criterion, mean (SD)		232.8 (44.2)		234.9 (46.4)
	Mean difference (SD)		−104.6 (83.8)		−29.1 (80.2)
	% mean relative error (SD)		−41.9 (1.3)		−11.2 (30.8)
	% mean absolute error (SD)		41.9 (31.3)		26.7 (17.0)
	Pearson correlation		–.26		.22

**Figure 6:**
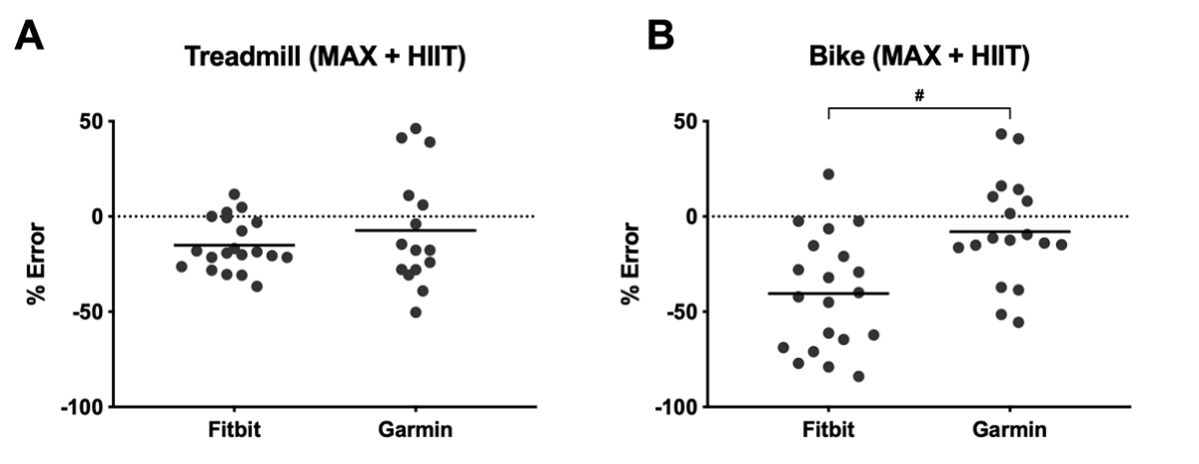
Percent relative error (RE) in energy expenditure (EE) during the VO_2_ peak test (MAX) and high-intensity interval training (HIIT) on the treadmill (A) and cycle ergometer (B) for Fitbit and Garmin. Negative bias in estimating EE is demonstrated by both devices during both modes of exercise, with the greatest mean error displayed by Fitbit during MAX and HIIT performed on the cycle ergometer. The horizontal lines represent the mean. # indicates *P*<.001.

MAPE (SD) for Garmin and Fitbit was 27.0% (SD 21.8) and 25.1% (SD 17.3), respectively. The lowest mean error in measuring EE was observed during ADL (−8.8% [SD 29.2]) for Fitbit and MAX-C (−4.5% [SD 25.3]) and HIIT-T (−4.7% [SD 29.3]) for Garmin. The highest error was observed during MAX-C (−39.1% [SD 30.6]) and HIIT-C (−41.9% [SD 31.3]) for Fitbit and resistance (21.0% [SD 35.7]) for Garmin. Figure 6 shows the relative error in EE for Fitbit and Garmin during all pooled treadmill and pooled cycle ergometer activities as scattered dot plots.

**Figure 7:**
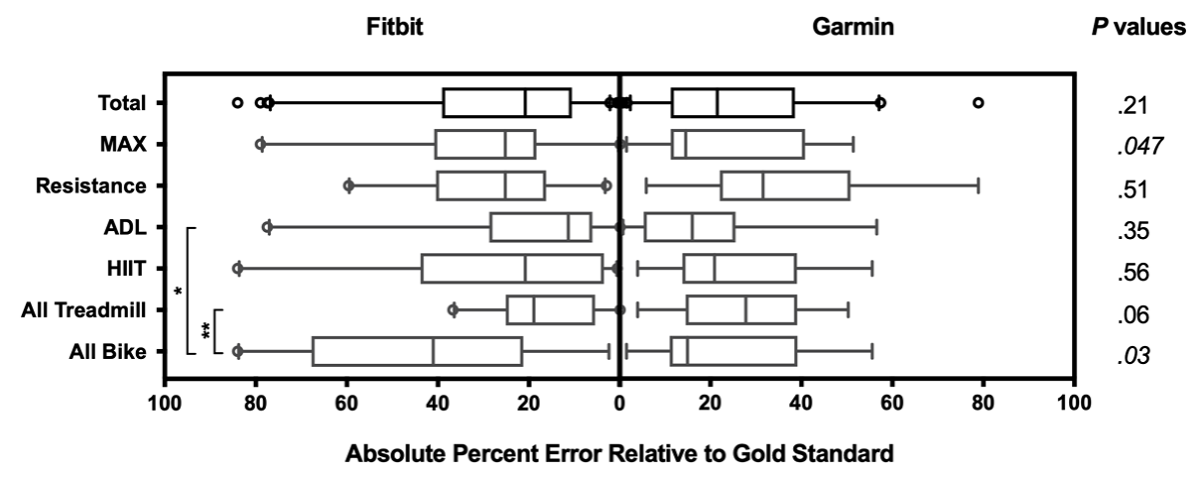
Absolute percent error in energy expenditure (EE) across different exercise modalities for Fitbit and Garmin. Each box-whisker plot consists of a box that extends from the 25% to the 75% quartile, with a line in the middle of the box representing the median (50% quartile). Each box has error bars that extend to the 5% and 95% quartiles, with outliers displayed with open circles. The *P* values listed on the right side display the difference in absolute percent error for EE between Fitbit and Garmin during each activity with italics indicating statistical significance. Asterisk and double asterisks indicate *P*=.02 and *P*=.003, respectively. ADL: activities of daily living, HIIT: high-intensity interval training.

Both Fitbit and Garmin demonstrated negative bias when activities were performed on the treadmill (Fitbit: −15.1% [SD 13.5], Garmin: −7.4% [SD 30.1]; *P*=.18). For activities performed on the cycle ergometer, both devices displayed negative bias, but there was significantly higher mean error for Fitbit compared with Garmin (Fitbit: −40.5% [SD 30.2], Garmin: −7.9% [SD 27.6]; *P*<.001). Figure 7 shows the absolute percent error in EE during each activity as box-whisker plots for Fitbit and Garmin, compared with Cosmed-derived EE.

Garmin was significantly more accurate than Fitbit at estimating EE during MAX (Fitbit: 31.5% [SD 21.5], Garmin: 22.9% [SD 16.8]; *P*=.047) and all cycle ergometer activities (Fitbit: 42.7% [SD 26.8], Garmin: 22.8% [SD 16.6]; *P*=.03). Fitbit was significantly more accurate than Garmin at estimating EE during ADL (ADL: 20.9% [SD 21.8], ergometer: 42.7% [SD 26.8]; *P*=.02) and all treadmill activities (Treadmill: 16.9% [SD 10.9], ergometer: 42.7% [SD 26.8]; *P*=.003) compared with all activities performed on the cycle ergometer.

### Spurious Heart Rate Measurements

**Figure 8:**
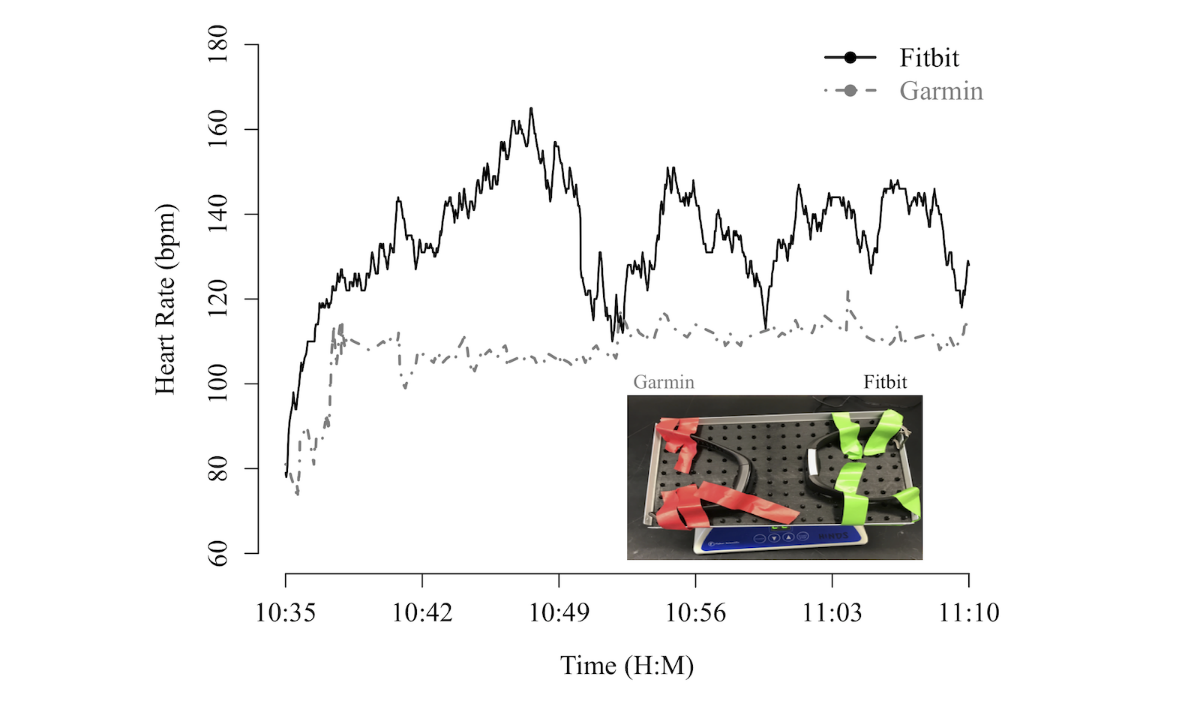
Spurious heart rate measured by the Garmin and Fitbit devices when placed on a shaker device (image of the experimental setup in the inset). H:M is hours:minutes.

During the early-phase testing of these devices, it was discovered that both devices would produce spurious HR measurements during periods of nonwrist use, such as when devices were stored in a backpack during commute. PPG sensors use a light source, commonly a group of light emitting diodes, to illuminate the tissue of the wrist, and the HR measurement is based on the differential reflection of the light as measured by the photodetector in response to the pulsatile nature of the blood perfusion in the superficial vessels. Under these working principles, if there is no light reflection from the surface, we suspected that the devices report HR measurements even if they are not *on body* (ie, spurious results). We performed a simple laboratory experiment to confirm this. Using a standard bench-top variable speed laboratory nutator (Fisher Sci # S06622), we simulated 3D wrist rotating motion at a fixed speed (22 rpm), and we recorded spurious HR results from both Garmin and Fitbit devices. The data and the experimental picture are shown in Figure 8.

## Discussion

### Principal Findings

This study examined the accuracy of 2 common wrist-worn, consumer-grade activity monitors for estimating HR and EE during a variety of nonsteady state activities. Similar to previous studies [13,28,30-32], we found reasonable accuracy in HR and EE estimations for these 2 devices under certain exercise conditions. Our findings are also in agreement with several prior studies that looked at HR and EE estimates across many different devices [16,17]; however, these 2 prior studies took measurements only at steady state conditions once HR had stabilized. A recent review by Bunn et al [33] showed that EE was generally underestimated by physical activity devices and that HR measurements were generally more accurate at rest or on a cycle ergometer as compared with treadmill. Dondzila et al [34] also looked at the Fitbit Charge HR and found that with aerobic exercise under laboratory conditions, the Fitbit Charge HR underestimated the HR compared with a Polar chest strap, with higher error at slower speeds. Jo et al [35] compared the Basis Peak and the Fitbit Charge HR with ECG and also found a negative bias of HR with respect to ECG measurements (−4.9 bpm for the Basis and −12.7 bpm for the Fitbit). In results presented in this paper, HR and EE measured by both the Garmin and Fitbit devices during the resistance exercise were similar to the measurements reported by Boudreaux et al [24]. Although, the resistance exercises were different, the intensity of the exercises was similar. There are 3 novel contributions from this study. First, we report HR accuracy in these activity monitors in modes not tested previously (eg, ADL and HIIT). Second, we show that HR accuracy as measured by these activity monitors is acceptable during low-intensity activities and high-intensity activities with repetitive wrist motion but that HR accuracy is poorer when there is no repetitive wrist motion and when any activity is at a high intensity (ie, ≥70% of maximal aerobic capacity). Prior research has suggested that PPG sensors used to measure the HR are liable to poor accuracy during activities with increased physical exertion or activities involving repetitive contractions of forearm skeletal muscles [36-38]. It has been suggested that during activities involving sustained muscle contractions or higher intensity exercises, the contact between the device’s PPG sensor and skin is decreased, leading to a disruption in the signal quality and causing poor quality data [36,37]. Third, we show that HR, as measured by the Garmin, is significantly improved when the device is in the activity mode setting. As the HR measurement algorithm is proprietary to Garmin, we do not know why the accuracy is worse when activity mode is not indicated. It appears that the watch uses different HR measurement algorithms depending on the activity mode selected. It may be that the activity mode algorithms implement less smoothing than the nonactivity mode algorithm and are thereby designed to respond faster to rapid HR changes.

Although both activity monitors showed reasonable accuracy in HR, we did see differences between the 2 activity monitors in EE estimates across all activities, and both activity monitors correlated poorly with indirect calorimetry measures of EE. It is unclear why we found poor estimation of the EE. EE values are dependent on many anthropometric characteristics of the subject as well as the HR measurements [14]. We assume that the EE estimations provided by these devices are also utilizing this information, but these calculations are proprietary. According to the manufacturers, Fitbit’s EE estimate includes both active calories and the basal metabolic rate (BMR), whereas Garmin only reports active calories without BMR. Even with the inclusion of BMR in EE estimates, Fitbit still displayed a greater negative bias during most activities compared with Garmin. If EE estimates by Garmin included BMR, there would likely be greater accuracy in the EE values reported by these devices. At the time of testing, these activity monitors provide different ways to indicate the various types of activity such as running, stationary bike, strength training and “other,” but there is not a clear indication for activities such as HIIT. Perhaps this is the reason for the high error rate recorded during these types of activities. As these consumer devices are constantly improved by the respective companies, the algorithms estimating EE should be improved or personalized to provide more accurate estimates. As these wearables transition from consumer reporting tools to clinical monitoring devices, a higher level of accuracy and precision is required. Clearly, the algorithms running on these wearables that estimate HR and EE are proprietary and can change without warning from the manufacturers, which poses further challenges for those wanting to integrate these devices into medical products. The onus of integrating these devices and assessing the level of accuracy and precision needed to make drug dosage decisions rests in the hands of those designing and evaluating medical algorithms.

Integrating these activity monitors into medical systems such as type 1 diabetes decision support systems or automated drug delivery systems in the future will require high fidelity data both from the HR signal and the EE estimates. The findings from this study point to shortcomings that could arise in both detecting activity and distinguishing the type of activity based on the HR signal. Although the mean error of the HR measurement was within the acceptable range for both devices, the range of the error was wider than anticipated. This issue and the inaccuracies associated with the EE data could lead to issues with estimating the intensity of the activity accurately. Additionally, short nonsteady state exercises such as a 10-second maximal sprint have been shown to influence the rapid change in glucose response to aerobic exercise [39], but findings from this study indicate that detecting these quick nonsteady exercises might be challenging for activity monitors to capture. We found spurious HR measurements when the activity monitor device is not worn on the wrist. Integration of these devices into a life-supporting drug delivery system must account for an on-wrist/off-wrist detection algorithms, which are currently not a part of the activity monitors evaluated. Another feature that could be integrated with further evaluation into a medical system is the exercise detection that is available on these devices. The Garmin device performed better when the exercise type was indicated through a button press on the watch. Future versions of these wearables are integrating automated exercise detection, and this is an area that should be further researched in terms of accuracy. Finally, if physical activity data are to be properly incorporated into medical systems including real-time drug delivery systems, access to the data in near real time (eg, every 5 min) would be important. In the automated insulin dosing scenario, for example, decisions would need to be made at the onset of exercise to prevent exercise-induced hypoglycemia. Currently, neither of these watches provide real-time access to their data streams. An approach to overcome some of the challenges associated with exercise detection and accuracy of detection would be to alert the individual before exercise dosing decisions are made. Effective integration of activity monitors is an active area of research in the medical community, and the findings from this study point to both the abilities and challenges associated with real-time monitoring and integrating into medical systems.

### Limitations

Our study has a limitation in that we only tested 2 popular consumer-grade devices. The choice was based on the ubiquity of these sensors in the market, affordability, and potential to be easily integrated into existing medical system architectures through, for example, an API. Our current data and interpretations may be limited as we did not account for the skin color in our study. It has been reported that skin color could influence the accuracy of the HR measurement [16], and future studies should report the Fitzpatrick skin tone scale to account for this limitation. Another limitation of our study is that exercise was conducted in a laboratory setting as opposed to the real world. However, we attempted to capture several real-world ADLs to minimize this limitation, though these activities were also recorded within a lab. It would be important to do further investigations in real-world settings to corroborate our results. Another limitation was that HR measurements from the wearable devices were not compared against a true gold standard such as ECG.

### Conclusions

We conducted a thorough assessment of 2 of the most popular low-cost consumer wrist-worn activity monitors during multiple exercise modalities and during daily activities. We found that during steady-state activities and during low-intensity activities, the HR measurements were within an acceptable error range (5%) but less accurate during higher intensity more dynamic activities that do not involve wrist motion. The EE estimates provided by these devices were inaccurate during all activities.

## Abbreviations

ADLs: activities of daily living
BMR: basal metabolic rate
BTLE: Bluetooth low energy
EE: energy expenditure
HIIT: high-intensity interval training
HIIT-C: high-intensity interval training-Cycle ergometer
HIIT-T: high-intensity interval training-Treadmill
HR: heart rate
MAPE: mean absolute percent error
MAX-C: MAX-Cycle ergometer
MAX-T: MAX-Treadmill
MET: metabolic equivalent
PPG: photoplethysmography
RPE: Rating of Perceived Exertion
VO_2_: oxygen consumption
RE: mean relative error
